# Impact of pressure ulcer prevention knowledge and attitude on the care performance of long-term care facility care workers: a cross-sectional multicenter study

**DOI:** 10.1186/s12877-022-03702-3

**Published:** 2022-12-21

**Authors:** Sae-Beul Lee, Hyang-Yuol Lee

**Affiliations:** 1 Graduate School, The Catholic University of Korea, Seoul, Republic of Korea; 2 College of Nursing, The Catholic University of Korea, 222 Banpo-daero, Seocho-gu, Seoul, 06591 Republic of Korea

**Keywords:** Pressure ulcer, Nursing homes, Knowledge, Attitude, Work performance

## Abstract

**Background:**

The Long-Term Care Insurance Act in the Republic of Korea has enabled the elderly population to receive benefits through the long-term care system since July 2008. Because one nurse or nursing assistant is assigned to 25 elderly persons and one care worker is assigned to 2.5 elderly persons in long-term care facilities, registered nurses should educate care workers to participate in pressure ulcer prevention activities. This descriptive study investigated the effect of the knowledge and attitude related to pressure ulcer prevention on care performance.

**Methods:**

Data were collected from February 20 to December 15, 2021 using a structured questionnaire targeting 165 care workers in four long-term care facilities located in I-city and Y-gun, Gyeongsangbuk-do. The questionnaires of the knowledge, attitude, and care performance developed for nurses were modified to survey the care workers. The content validity was verified on a 4-point scale by 10 clinical experts. A preliminary survey was conducted for 30 care workers, and the contents of the modified questionnaire were further revised. Data analyses were performed by t-test, one-way ANOVA, Scheffé test, Pearson’s correlation coefficient, and a multiple linear regression modeling using the SPSS/WIN 25.0 program.

**Results:**

Care performance on pressure ulcer prevention had a significant positive correlation with knowledge (*r* = 0.692, *p* < .001), attitude (*r* = 0.426, *p* < .001), work experience (*r* = 0.760, *p* < .001), amount of experience (*r* = 0.712, *p* < .001), and the number of training sessions received (*r* = 0.551, *p* < .001). In multiple regression modeling, work experience (β [standardized coefficient beta] = 0.534, *p* = .000), knowledge (β = 0.323, *p* = .000), and attitude (β = 0.103, *p* = .049) related to pressure ulcer prevention were identified as variables significantly affecting care performance. The regression model explained 65.4% with 5 independent variables.

**Conclusion:**

To prevent bedsores in long-term care facilities, it is necessary to educate care workers regularly about pressure ulcer prevention. In addition, clinical guidelines could help standardize the pressure ulcer prevention work of caregivers, strongly regulating their practice in all long-term care facilities and monitoring bedsore prevention regularly.

## Introduction

Korea effectively became an aging society in 2018 when the elderly population reached 14.3% of the total population [1]. Increased life expectancy, chronic diseases, and the burden of medical expenses are recognized not only as problems of individuals and households but also as social and national responsibility, and as a result, long-term care insurance was implemented starting in July 2008 in accordance with the Long-Term Care Insurance Act [2]. This enabled more elderly people to receive benefits through the long-term care system. Welfare facilities for the elderly include residential facilities, medical facilities, leisure facilities, in-home care facilities, and elderly protection agencies [3]. In this study, elderly nursing facilities—one type of medical welfare facility for the elderly, excluding long-term care group homes from the medical welfare facilities for the elderly—were investigated. The commonly used term “long-term care facility” was used instead of “elderly nursing facility” where mainly elderly people who require moderate or higher long-term nursing services are admitted and live. According to the Welfare of Senior Citizens Act, the objective of long-term care facilities is to provide the conveniences and accommodations necessary for everyday life, including meals and care among others by admitting the elderly who require assistance due to significant impairments both physically and mentally caused by geriatric diseases such as dementia and stroke [4].

Care workers are certified after successfully passing a qualification test at a professional training institution and provide professional care including physical and housekeeping activities for the elderly [5]. According to the Elderly Welfare Act, 1 nurse or nursing assistant is assigned to 25 elderly persons and 1 care worker is assigned to 2.5 elderly persons in long-term care facilities [6]. Materials from the National Health Insurance Service show that a total of 111,895 elderly people used long-term care facilities nationwide in 2020 [7] and there were 76,011 care workers and 8932 nursing assistants working of long-term care facilities in 2020 [8]. Care workers take up a large portion of the staff and personnel of long-term care facilities and provide direct services like bodily care and overall assistance in everyday life activities; thus, care workers play an important role in the service quality of long-term care facilities [9]. Care workers, as non-medical personnel, comprise the major workforce and directly care for elderly patients in long-term care facilities and perform direct pressure ulcer prevention activities on duty, whereas nurses manage actual pressure ulcers as medical personnel. So, it is critical to educate care workers to perform those activities well.

In Korea, care workers require national qualification but cultivating care workers with expertise is difficult due to the short training period of 240 hours and a high pass rate of 90% or higher because of the test’s low difficulty level [10]. A previous study on the level of knowledge of pressure ulcers by care workers working in long-term care hospitals and care homes gave a low score of 12.84 points out of 24 points [11]. Also, a previous study that investigated the relationship between knowledge and performance for 90 care workers in 3 long-term care facilities resulted in a relatively high score of 5.59 points out of 7 points for the knowledge level, but the study stated that determining whether care workers are aware of the mechanism of pressure ulcer occurrence and importance of pressure ulcer prevention methods as well as such education is necessary [12]. Although occupational competency strengthening programs are carried out in individual long-term care facilities after acquiring qualification, it is not a systematic training system, and although the need for the strengthening of care worker cultivation programs and systematic occupational training was presented through various previous studies, care workers in Korea lack an occupational training system in comparison to care workers of other countries [13]. Due to the lack of pressure ulcer prevention-related expertise by care workers, pressure ulcers for the elderly become the main burden of diseases and are related to the increase in pain, discomfort, psychological stress and depression, risk of infection, complications, and mortality [14]. Moreover, this leads to increases in medical expenses both individually and nationally. Thus, additional training is necessary to further improve care workers’ expertise.

Korea’s care worker qualification system has a direct impact on the quality of the service provided by care workers and related discussions have been actively conducted [15–18]. For care workers in Japan, a requirement of certification is a high school diploma or an equivalent education level [19], and care workers in Germany must complete a total of 4600 hours of training and education [20]. On the other hand, there are no qualification requirements, such as age or education level for care workers in Korea; qualification for certification is given to anyone taking 240 hours of training and education are required [19]. It is thought that there were no qualification requirements for care worker training from the beginning because the demand for care workers was urgent and high. However, such cultivation of manpower inevitably leads to a decrease in the quality of nursing care by caregivers.

Pressure ulcers are commonly called pressure ulcers, bedsores, and decubitus ulcers, and are defined as localized damage to the skin or tissue under the skin due to continuous pressure on bone-protruding areas [21]. Pressure ulcers are caused by restricted movement, a lack of moisture, degradation of sensory perceptions, diabetes, and malnutrition, and the elderly are relatively more vulnerable to pressure ulcers [22]. The prevalence of pressure ulcers among the elderly using long-term care facilities in Korea is 4.3 ~ 9.8% [23, 24], and the prevalence of pressure ulcers in overseas studies varied from 4.03 ~ 14.42% [25] and 16.7 ~ 43.3% [26].

Regarding pressure ulcer prevention, numerous studies have examined nurses [27, 28]; however, research of care workers is limited to the studies by Kim et al. [9] and Choi et al. [12]. Kim et al. investigated the relationship between pressure ulcer prevention training and the knowledge level of pressure ulcer prevention for 81 care workers working in long-term care hospitals and care homes in 2012, and this study revealed that care workers who received the pressure ulcer prevention training exhibited a higher level of knowledge [9]. Choi et al. surveyed 90 care workers working in 3 long-term care facilities in 2016 and showed that the level of pressure ulcer prevention knowledge must be improved to enhance the performance of pressure ulcer prevention [12]. Previous studies by Kim et al. and Choi et al. of care workers investigated the knowledge level and relationship between knowledge and care performance, respectively [9, 12]. In this study, care workers working in long-term care facilities were surveyed to examine the relationship between knowledge, attitude, and care performance. Although one study investigated pressure ulcer knowledge, attitude, and performance of nurses at long-term care facilities for the elderly and one study examined the relationship between the attitude towards pressure ulcer prevention and care performance of nurses [29], research on care workers is lacking. Knowledge is also a significant prediction factor related to pressure ulcer prevention, so knowledge was included in this study and care workers were selected as the subjects to carry out a different and original study. Because previous studies have surveyed care workers limited to specific regions, research that investigated more subjects across wider areas and regions is necessary for the generalization of the research results. The subjects of this study were care workers of long-term care facilities located in the urban region of I City and countryside region of Y-gun Gyeongsangbuk-do Province, and a greater number of research subjects were involved in comparison to previous studies for the greater facilitation of research result applications to care workers. Furthermore, this study aimed to provide basic data and material for the future development of training and education programs for care performance improvement of care workers regarding pressure ulcer prevention.

The objectives of this study were to determine the level of pressure prevention knowledge, attitude, and care performance by care workers at long-term care facilities and identify the impact of pressure ulcer prevention knowledge and attitude on care performance.

## Methods

### Research design

This study is a descriptive survey study conducted to investigate the pressure ulcer related knowledge, attitude, and care performance of care workers working at long-term care facilities and determine the impact of pressure ulcer prevention knowledge and attitude on care performance.

### Research subject

The subjects of this study were care workers who provide services directly to the elderly at 4 long-term care facilities registered under the National Health Insurance Service. Three facilities are located in I City and one is in Y-gun of Gyeongsangbuk-do Province. Using purposed sampling, care workers with work experience of at least 1 month at the long-term care facility and those who understood the objectives of this study and agreed to participate in this study were recruited as the subjects of this study.

The number of subjects was calculated using the G*power 3.1.9.7 software through multiple regression analysis. The minimum sample size necessary was 147 people calculated based on a medium effect size of 0.25, significance level of 0.05, power of 0.90, and prediction factors including 2 major variables (pressure ulcer prevention related knowledge and attitude) and 8 general characteristics (age, gender, education level, work experience at long-term care facilities, work location, amount of pressure ulcer experience, completion of pressure ulcer prevention related training after receiving care worker certification, and number of training sessions and average time duration of 1 session when training was completed). Taking into consideration the dropout rate of 10%, the minimum sample number was 161 people, and the survey was conducted by a total of 165 people. There were no incomplete responses, and the responses of 165 people were used in the final data analysis.

### Research tool

In this study, a survey was carried out for data collection, which included a total of 48 items composed of 8 general characteristics items, 15 items regarding pressure ulcer prevention related knowledge, 9 items regarding pressure ulcer prevention related attitude, and 16 items regarding pressure ulcer prevention related care performance. The selection of survey items was carried out from May 7, 2020, to August 25, 2020, where a preliminary survey was conducted for 10 care workers and the terminology and wording were modified for better understanding by care workers. Then, the content validity index (CVI) was used to analyze whether the survey was appropriate in determining the pressure ulcer knowledge level of care workers, whether the survey uses terminology and words that care workers can understand, whether the item difficulty is appropriate to reflect the characteristics of the group, and whether the items and responses are accurate from the perspective of clinical experts. Data were collected from 2 home healthcare nurses, 2 gerontological nursing doctors, 5 social welfare master’s graduates, 1 contract doctor, and 10 clinical experts all with at least 5 years of work experience at long-term care facilities. The CVI tool employed a 4-point scale from “not relevant” (1 point), “cannot determine the relevance without modification of the item” (2 points), “relevant but requires slight changes to the item” (3 points), and “highly relevant” (4 points), and the CVI value was calculated for each item where items with a CVI of less than 0.8 were removed. As a basis for content validity, Lee and Shin [30] determined that studies that selected items with CVI values of 0.8 or higher were dominant at 58.1%, and based on this, the criterion for CVI was set to 0.8.

#### Subjects’ general characteristics

The subjects’ general characteristics that were investigated included age, gender, education level, work experience at long-term care facilities, work location, amount of pressure ulcer experience, completion of pressure ulcer prevention related training after receiving care worker certification, and number of training sessions and average time duration of 1 session when training was completed.

#### Pressure ulcer prevention related knowledge

The pressure ulcer prevention related knowledge referenced the tool developed by Beitz, Fey, and O’Brien [31] for nurses, which was modified, supplemented, and translated by Lee [32], who added the following subcategories: knowledge on pressure ulcer occurrence risk factors, pressure ulcer conditions, and pressure ulcer treatment methods. Nineteen items were selected pertaining to the actual services provided by care workers at long-term care facilities. Among these items, 4 items with CVI values of 0.8 and lower were deleted so that the knowledge survey of this study comprised a total of 15 items. The tool’s maximum score was 15 points and minimum score was 0 points, where a right answer was 1 point and wrong answer and “do not know” were 0 points. A higher score signifies a higher knowledge level. Cronbach’s alpha or reliability of the tool was 0.66 at the time of development [31]; it was 0.72 in the study by Lee, whereas it was 0.789 in this study.

#### Pressure ulcer prevention related attitude

With regard to the attitude towards pressure ulcers, Moore and Price [33] developed 4 subcategories for nurses: pressure ulcer prevention, pressure ulcer behavior, barriers towards pressure ulcer prevention, details about your practice. Among these subcategories, Seo [11] translated the pressure ulcer prevention category. The tool translated by Seo [11] was used to determine, modify, and supplement items appropriate for long-term care facilities, and ultimately 10 items were used. Among the 10 items, 1 item with a CVI of 0.8 or lower was removed and the attitude survey of this study comprised 9 items. This tool employed a 5-point scale with responses of “strongly disagree” (1 point), “disagree” (2 points), “neither agree nor disagree” (3 points), “agree” (4 points), and “strongly agree” (5 points). A minimum of 9 points and maximum of 45 points are possible, and a higher score signifies a more positive attitude towards pressure ulcers. In the 9 items, items 3, 4, 7, and 8 were scored in reverse order and summed for utilization. Cronbach’s alpha or reliability of the tool was 0.78 in the study by Kang and Kim [34], whereas it was 0.796 in this study.

#### Pressure ulcer prevention related care performance

For pressure ulcer prevention care performance, Kwon [35] developed 21 items by referencing the 3-point scale tool for nurses based on the pressure ulcer prevention and intervention guidelines of the US Agency for Healthcare Research and Quality (AHCPR). In this study, a total of 18 items were composed after selecting items appropriate for observing the care performance of care workers. To minimize limitations in the self-reporting survey responses, the researcher of this study evaluated some items by directly observing pressure ulcer prevention related care performance. All items exceeded the CVI value of 0.8, so all 18 items were going to be maintained, but keeping items 2 and 8 resulted in a reliability of 0.403, whereas removing the items resulted in a reliability of 0.711, so the 2 items were removed. The original tool used a 3-point scale, but it was rearranged in this study to use the more generally used 5-point scale. The responses available were “strongly disagree” (1 point), “disagree” (2 points), “neither agree nor disagree” (3 points), “agree” (4 points), and “strongly agree” (5 points). A minimum score of 16 points and maximum score of 80 points are possible, and a higher score signifies greater pressure ulcer prevention related care performance. Cronbach’s alpha or reliability of the tool was 0.91 in the study by Kwon [35], whereas it was 0.711 in this study.

### Data collection

For the data collection, the researcher explained the objectives and method of this study to the superintendent or director of the long-term care facilities where the study subjects work, and the study objectives were explained to the subjects (care workers) after receiving permission for data collection. The survey, along with a small token of appreciation, were distributed to the subjects only when the subjects agreed to participate in the study, and the completed surveys were placed in a yellow envelope right away and sealed.

Due to the risk of COVID19 infection in long-term care facilities, 30 surveys were collected from February 20 to March 1, 2021; 60 surveys were collected from September 18 to September 30, 2021; and 75 surveys were collected from November 1 to December 15, 2021. One participant performed only one survey during the data collection period. Of the 4 long-term care facilities, 50 respondents were recruited from the first institution in I city with 100 beds, 92 patients, and 44 care workers. Fifty-three participants were recruited from the second institution in I city with 160 beds, 132 patients, and 60 care workers. Thirty-nine respondents were recruited from the third institution in I city with 83 beds, 83 patients, and 40 care workers. Finally, 23 participants were recruited from the fourth institution in Y-gun of Gyeongsangbuk-do province, which had 72 beds with 60 patients and 26 care workers.

### Ethical considerations

With regard to the content and methods of this study, approval was obtained from the university’s Institutional Review Board (IRB) (IRB:MC20QISI0126). The researcher explained to subjects that they could withdraw from participating in the study at any time and the collected data were confidential. The surveys were collected right away after completion and sealed in a yellow envelope for confidentiality. The surveys were stored in a drawer with a locking device. The collected data are planned to be stored for 3 years and will be destroyed afterwards.

### Detailed objectives

The detailed objectives of this study were as follows.

1. Investigate the general characteristics of long-term care facility care workers and the average differences in knowledge, attitude, and care performance according to the general characteristics.
2. Investigate the level of pressure ulcer prevention knowledge of care workers at long-term care facilities.
3. Investigate the pressure ulcer prevention attitude of care workers at long-term care facilities.
4. Investigate the level of care performance related to pressure ulcer prevention of care workers at long-term care facilities.
5. Investigate the relationship between pressure ulcer prevention knowledge, attitude, and care performance of care workers at long-term care facilities.
6. Investigate the impact of pressure ulcer prevention knowledge and attitude on the care performance of care workers at long-term care facilities.

### Data analysis

The data collected in this study were analyzed using SPSS/WIN 25.0 program.

1. Subjects’ general characteristics were analyzed by real number, percentage, average, and standard deviation.
2. The t-test, one-way ANOVA, and Scheffé test were used to analyze the differences in pressure ulcer prevention related knowledge, attitude, and care performance according to the subjects’ general characteristics.
3. Subjects’ pressure ulcer prevention related knowledge, attitude, and care performance were analyzed by average and standard deviation.
4. The relationships between subjects’ pressure ulcer prevention related knowledge, attitude, and care performance were analyzed using the Pearson’s correlation coefficient.
5. To determine the effects of pressure ulcer prevention related knowledge and attitude on subjects’ care performance, multiple linear regression was performed by controlling the general characteristics that could have a significant impact.

## Results

### Subjects’ general characteristics

The average age of the study subjects was 64 years old, and the subjects were 100% female. The education level of elementary school or lower was highest at 66.7%. The location of the care facility where the subjects currently work was mainly in the Seoul metropolitan area (86.1%), and 83.0% of subjects responded that they received pressure ulcer related training. The average number of pressure-ulcer-occurrence experience was 4.26 ± 5.05 times (range 0 ~ 20). The number of training sessions was 3.43 ± 2.85 (range 1 ~ 15) and the training time duration per session was around 18.97 ± 10.05 minutes (range 5 ~ 60). Depending on whether the subjects received pressure ulcer prevention related training, a significant difference between the pressure ulcer prevention related knowledge (t = 4.55, *p* = .000) and care performance (t = 4.31, *p* = .000) was observed (see Table 1).

**Table 1:** Participants’ general characteristics (*N* = 165)

Characteristics	Categories	Total		Pressure ulcer prevention knowledge		Attitude toward pressure ulcer prevention		Pressure ulcer prevention performance	
		*n* (%)	M ± SD	M ± SD	t or F(***p***)	M ± SD	t or F(***p***)	M ± SD	t or F(***p***)
Age (yr)	< 65	85 (51.5)	64.09 ± 4.50						
	≥65	80 (48.5)							
Gender	Woman	165 (100)							
	Man	0 (0.0)							
Educational status	≤Elementary school	110 (66.7)		7.15 ± 2.73	0.57 (.566)	26.77 ± 3.91	0.07 (.933)	47.40 ± 8.12	1.86 (.160)
	Middle school	47 (28.5)		7.06 ± 2.53		26.61 ± 3.35		45.97 ± 7.06	
	High school	7 (4.2)		6.13 ± 1.36		27.12 ± 4.79		42.50 ± 2.97	
	≥University	1 (0.6)							
Care-worker experience (year)	≤1	49 (29.7)	33.21 ± 31.67						
	1–3	64 (38.8)							
	3–5	21 (12.7)							
	5–7	15 (9.2)							
	7–9	10 (6.0)							
	≥9	6 (3.6)							
Current workplace	Metropolitan region	142 (86.1)		7.02 ± 2.64	−0.70 (.485)	26.59 ± 3.87	−1.24 (.217)	46.26 ± 7.20	−1.61 (.119)
	Rural area	23 (13.9)		7.43 ± 2.55		27.65 ± 3.12		49.78 ± 10.03	
Experience frequency of pressure ulcer	0	38 (23.0)	4.26 ± 5.05						
	1	29 (17.6)							
	2–5	47 (28.5)							
	6–10	41 (24.8)							
	11–15	2 (1.2)							
	15–20	8 (4.8)							
Educational experience	Yes	137 (83.0)		7.39 ± 2.66	4.55 (.000)	26.94 ± 3.78	1.48 (.142)	47.56 ± 7.99	4.31 (.000)
	No	28 (17.0)		5.53 ± 1.79		25.78 ± 3.71		42.82 ± 4.55	
Number of times for education on Pressure ulcer	0	28 (17.0)	3.43 ± 2.85						
	1–5	108 (65.5)							
	6–10	28 (17.0)							
	≥11	1 (0.6)							
Education time (minute per 1 education) (*n* = 137)	≤10	49 (35.8)	18.97 ± 10.05						
	11–20	66 (48.2)							
	21–30	13 (9.5)							
	≥31	9 (6.5)							

*M* Mean, *SD* Standard Deviation

### Pressure ulcer prevention related knowledge of care workers

The average score for subjects’ pressure ulcer prevention related knowledge was 7.08 ± 2.62 points (range 2 ~ 14) out of a total of 15 points. The lowest correct answer rate was in the order of “pressure ulcers do not occur from sitting down for too long (12%)” and “treatment of pressure ulcers can be accelerated by massaging areas of reddened skin (13%).” The highest correct answer rate was “the conditions of the skin should be checked periodically (89%)” and “changing the positions of those with the possibility of pressure ulcer occurrence should be done at least once every 2 hours (89%)” (see Table 2).

**Table 2:** Pressure ulcer prevention knowledge of care workers (*N* = 165)

No.	Knowledge of pressure ulcer prevention	Correct answer
		*n* (%)
K14	The skin condition of the elderly should be checked periodically.	147 (89)
K15	Changes in the position of elderly with the possibility of developing pressure ulcers should be made at least once every 2 hours.	147 (89)
K12	A little humidity, cleanliness and warmth are effective in healing pressure ulcers.	140 (84)
K1	People who are unconscious have a high incidence of pressure ulcer.	120 (72)
K4	Lifting the patient using a quilt or the like can reduce friction and cohesion (pushing force).	79 (47)
K2	Lying in the same position for more than 30 minutes can damage tissue due to pressure.	76 (46)
K3	When taking a positioning (lying on the side), it is necessary to maintain 30 degrees.	75 (45)
K7	It is desirable that the surface of pressure ulcers be kept dry at all times.	41 (24)
K13	Even in a healthy person, if you stay in the same position for 2–4 hours, pressure ulcers will occur.	39 (23)
K10	Air beds, parchments (soft leather) are effective in reducing pressure in bone protrusions.	35 (21)
K5	Weight should be taken into account when assessing the risk of developing pressure ulcer.	34 (20)
K11	Pressure ulcers occur because of the suppression.	34 (20)
K6	Low body weight is a risk factor for the development of pressure ulcer.	27 (16)
K8	Massaging the reddened area of the skin can promote pressure injuries’ healing	22 (13)
K9	Sitting for a long time does not mean that pressure ulcer occur.	21 (12)

### Pressure ulcer prevention related attitude of care workers

The average score for subjects’ pressure ulcer prevention related attitude was 26.75 ± 3.79 points (range 19 ~ 36) out of a total of 45 points. The pressure ulcer prevention attitude score was lowest for “pressure ulcer prevention is of lower priority than other tasks (2.18±1.22 points)” and “prevention is of greater priority for pressure ulcers than treatment (2.33±1.17 points),” whereas the highest score was for “constant observation of conditions allows for the accurate identification of pressure ulcer risks (3.98±1.24 points)” (see Table 3).

**Table 3:** Care workers’ attitudes toward pressure ulcer prevention (*N* = 165)

No.	Attitude toward pressure ulcer prevention	M ± SD
A5	Constantly observing the condition can help you accurately identify the risk of developing pressure ulcers.	3.98 ± 1.24
A1	A lot of time is spent practicing interventions to prevent pressure ulcers.	2.77 ± 0.91
A3	I don’t worry about pressure ulcers prevention work. ^a^	2.70 ± 1.32
A6	Most pressure ulcers are preventable	2.58 ± 1.16
A2	Pressure ulcers are not often caused by elderly people who have recently been admitted.	2.56 ± 0.87
A9	Pressure ulcers risk assessments for elderly people living in long-term care facilities should be conducted on a regular basis.	2.42 ± 0.98
A7	I don’t have much interest in pressure ulcer prevention. ^a^	2.35 ± 1.13
A4	Pressure ulcers are a priority over treatment prevention. ^a^	2.33 ± 1.17
A8	Pressure ulcers prevention is a lower priority than other tasks. ^a^	2.18 ± 1.22
	Total score	26.75 ± 3.79

^a^reverse question

*M* Mean, *SD* Standard Deviation

### Pressure ulcer prevention related care performance of care workers

The average score of subjects’ pressure ulcer prevention related care performance was 46.76 ± 7.72 points (range 36 ~ 67) out of a total of 80 points. The lowest scores were “pay attention to the nutritional intake of people with pressure ulcers (1.65±0.80 points)” and “constantly check diapers and change diapers right away when identifying that the person has relieved him or herself (1.65±1.01points).” The highest score was “identify the next position to take every hour by checking the position change table (4.86±0.43 points)” (see Table 4).

**Table 4:** Pressure ulcer prevention performance of care workers (*N* = 165)

No	Performance of pressure ulcer prevention	M ± SD
P5	Look at the position change table to determine the position you need to take at each hour.	4.86 ± 0.43
P12	Change the position of elderly with pressure ulcers or possible pressure ulcers every 2 hours.	4.70 ± 0.51
P6	After the position change, it is recorded in the position change record sheet.	4.52 ± 0.82
P7	When moving elderly or changing their posture, they enter and move without dragging.	3.87 ± 0.74
P2	If there is a change in the skin condition of the elderly who have pressure ulcers or possible pressure ulcers, report them to the nursing department.	3.77 ± 0.81
P16	After the bath, the bite should be completely removed completely with a towel.	3.35 ± 0.97
P1	Periodically check the skin condition of elderly who may develop pressure ulcers or pressure ulcers.	2.88 ± 0.87
P14	Take care not to sit in the same position for more than 30 minutes in a wheelchair for elderly with possible pressure ulcers.	2.64 ± 1.01
P11	Check from time to time that the anti- pressure ulcers apparatus such as air mattresses (air beds) and air cushions work well.	2.54 ± 1.02
P10	If clothes, bed sheets, etc. are wet, replace them as soon as they are found.	2.46 ± 0.94
P8	Check diapers from time to time, and if the elderly urinate, change diapers as soon as you find them.	2.19 ± 1.05
P9	If the elderly is applying a restraint, periodically peel off to check the condition of the skin and massage it.	2.01 ± 1.03
P3	Maintain a position of 30 degrees when taking a position (lying on the side).^a^	1.91 ± 1.02
P13	Make sure that the hip area of the elderly is seated up to the back of the wheelchair.^a^	1.76 ± 1.03
P4	Support such as pillows and water bags in areas where the bones of the elderly and beds are in contact for a long time.^a^	1.65 ± 1.01
P15	Interested in the nutrition of elderly with pressure ulcers.	1.65 ± 0.80
	Total score	46.76 ± 7.72

^a^Questions observed by researchers to see whether they performed it correctly or not

### Correlation between the pressure ulcer prevention related knowledge, attitude, and care performance of care workers

The pressure-ulcer-prevention-related care performance was found to have a significant and positive correlation with pressure-ulcer-prevention-related knowledge (*r* = 0.692, *p* < .001), attitude (*r* = 0.426, *p* < .001), work experience (*r* = 0.760, *p* < .001), pressure ulcer experience number (*r* = 0.712, *p* < .001), and training session number (*r* = 0.551, *p* < .001) (see Table 5). Although age exhibited a significant and positive correlation with work experience (*r* = 0.337, *p* < .001), pressure ulcer experience number (*r* = 0.244, *p* = .002), training session number (*r* = 0.369, *p* < .001), and care performance (*r* = 0.174, *p* = .026), a significant correlation was not observed with pressure ulcer prevention related knowledge and attitude.

**Table 5:** Correlation among research variables (*N* = 165)

	1. Pressure ulcer prevention knowledge	2. Attitude toward pressure ulcer prevention	3. Pressure ulcer prevention performance	4. Care-worker experience (years)	5. Experience frequency of pressure ulcer	6. Education session numbers on pressure ulcer
1	1.00					
2	.323^**^	1.00				
3	.692^**^	.426^**^	1.00			
4	.664^**^	.422^**^	.760^**^	1.00		
5	.636^**^	.369^**^	.712^**^	.831^**^	1.00	
6	.484^**^	.170^*^	.551^**^	.685^**^	.662^**^	1.00

^*^p < .05, ^**^p < .001

### Impact on the pressure ulcer prevention related care performance of care workers

Table 6 shows the multiple linear regression result conducted to determine the impact of pressure ulcer prevention related knowledge and attitude as well as work experience, whether training was completed, and age as the control variable, which showed a significant effect on care performance among the care workers’ general characteristics. To verify the basic assumption of the multiple linear regression, the Durbin-Watson statistical analysis was used to test the self-correlation error, and the result was 2.034, close to the reference value of 2, so it was determined that there were no problems with self-correlation. Also, the variable tolerances (TL) of all 5 variables included in the model were lower than 0.1 (0.414 ~ 0.862) and the variation inflation factor (VIF) was lower than 10 (1.16 ~ 2.414), so there were no problems with multicollinearity. The multiple linear regression result showed that there was no significant impact of age and whether training was completed on care performance, but the factors of work experience (β [standardization coefficient beta] = 0.534, *p* = .000), pressure ulcer prevention related knowledge (β = 0.323, *p* = .000), and pressure ulcer prevention related attitude (β = 0.103, *p* = .049) were found to have a significant impact on the pressure ulcer prevention related care performance. The model’s explanatory power, including the 5 variables, was 65.4%. Work experience was the most impacting factor on pressure ulcer prevention related care performance.

**Table 6:** Ordinary least squares regression results predicting care performance on pressure ulcer prevention activities of care workers (*N* = 165)

Variables	B	SE	β	t	*p*
Constant	32.471	6.789		4.783	.000**
Age (yr)	−0.058	0.087	− 0.034	− 0.670	.504
Care-worker experience (years)	0.130	0.018	0.534	7.362	.000**
Education on Pressure ulcer (Yes)	1.150	1.030	0.056	1.116	.266
Pressure Ulcer Prevention Knowledge	0.949	0.186	0.323	5.096	.000**
Attitude Toward Pressure Ulcer Prevention	0.211	0.107	0.103	1.981	.049*

*R*^*2*^ = 0.654. Adjusted *R*^*2*^ = 0.643. Durbin-Watson = 2.034. *F* = 60.065, *p* < .001.

*p < .05, **p < .01, *SE* Standard Error

## Discussion

This study was carried out to determine the pressure ulcer prevention related knowledge, attitude, and care performance of care workers working in long-term care facilities and provide the basic data necessary to improve the quality of pressure ulcer prevention care performance.

The pressure ulcer prevention related knowledge was 7.08 ± 2.62 points out of a total 15 points. An existing tool was modified and supplemented for care workers, so a direct comparison with the scores of previous studies that investigated nurses is difficult, but overall, the score was low.

Among the items on pressure ulcer prevention related knowledge, the correct answer rate of the item “people who are semiconscious have a higher risk of pressure ulcers” was relatively high at 72%, which is similar to the results by Lee [32], Seo [11], and Kwon [35]. The correct answer rate of “when the patient is lifted using the bedsheet, friction and shearing force (sliding force) can be reduced” was 85% in the study by Lee [32] and 47% in this study, whereas the correct answer rate for the item of “weight needs to be considered when evaluating the risk of pressure ulcers” was 83% in the study by Lee [32] and 20% in this study. The higher correct answer rate for pressure ulcer prevention knowledge in the study by Lee [32] compared to this study for the same items could be due to the differences in working environments between university affiliated hospitals and long-term care facilities as well as the education level differences between nurses and care workers. A study of 234 people working at long-term care facilities revealed that the most important prediction factor of pressure ulcers was “friction and shearing force” [36], and the items related to this factor in this study were “30 degrees need to be maintained when in the position of lying to the side,” “when the patient is lifted using the bedsheet, friction and shearing force (sliding force) can be reduced,” and “pressure ulcers do not occur from sitting down for too long” and their correct answer rates were low at 45, 47, and 12%, respectively. Pressure ulcers can form when care workers who provide direct care for extended periods of time to the elderly in long-term care facilities do not have adequate knowledge; thus, sufficient training and education for care workers are necessary so care workers are knowledgeable regarding the prevention of pressure ulcers.

Among the pressure ulcer prevention related attitude items in this study, the average response of “pressure ulcer prevention is of lower priority than other tasks” was 2.18, a score that is closer to an unfavorable attitude. However, the study by Seo [11], which surveyed nurses in elderly nursing facilities revealed a favorable attitude with 0% responses for “strongly agree” and 6% for “agree.” The care workers surveyed in this study exhibited a negative attitude towards pressure ulcers compared to nurses. It was thought that nurses resulted in higher pressure ulcer prevention related attitude because they frequently experience pressure ulcers as they assist in pressure ulcer treatment in their work, therefore they can easily recognize the risks of pressure ulcers. By increasing the understanding of pressure ulcer prevention and recognition of its importance and necessity by care workers through pressure ulcer prevention training, attitudes are expected to become favorable with regard to pressure ulcer prevention. It is also necessary to subdivide the standardization and practice regulations for pressure ulcer prevention tasks so that the division of labor between nurses and care workers can be performed efficiently. However, because changes in attitudes are difficult to foster through training in a short period of time, it is necessary to continuously provide a differentiated training program according to care workers’ education level within the scope of their work.

Among the pressure ulcer prevention related care performance items in this study, the items of “pay attention to the nutritional intake of people with pressure ulcers” and “constantly check diapers and change diapers right away when identifying that the person has relieved him or herself,” which can be carried out properly only with sufficient knowledge of pressure ulcer prevention, were lowest with an average of 1.65 points. Items with relatively high care performance were “identify the next position to take every hour by checking the position change table” and “changing the positions of those with the possibility of pressure ulcer occurrence should be done at least once every 2 hours,” which had average scores of 4.86 and 4.70 points, respectively. The documentation and availability of a position change table and record at the 4 long-term care facilities where data collection was carried out in this study was considered to have affected the results. Specifying the order and time of position changes rather than leaving it up to the care worker and enforcing the recording of position changes every hour are necessary to improve the position change implementation for the prevention of pressure ulcers.

Scoring of the pressure ulcer prevention related knowledge, attitude, and care performance revealed results that were not high, suggesting care workers lacked pressure ulcer prevention related expertise. In this study, improving pressure ulcer prevention knowledge, instilling a favorable attitude toward pressure ulcers, and more work experience positively impacted the pressure ulcer prevention related care performance; thus, an official training program on pressure ulcers must be established for care workers.

The limitations of this study are as follows. This study was conducted with care workers working at long-term care facilities limited to a single region and the characteristics of each long-term care facility were not fully taken into consideration, so supplementary follow-up research is necessary. For the measurement of the pressure ulcer prevention related care performance, the collection of self-reporting survey responses and direct observation by the researcher were conducted, but the measurements of pressure ulcer prevention related knowledge and attitude relied on the self-reporting survey responses; thus, there are limitations in generalizing and applying the results of this study to all care workers.

Despite such limitations, this study is meaningful in terms of originality compared to previous research in that it was the first study to determine the effects of pressure ulcer prevention related knowledge and attitude on care performance for only care workers working at long-term care facilities. Existing tools were modified and supplemented for care workers, and the researcher directly observed care performance as well as collected the self-reporting survey responses for a more accurate measurement of pressure ulcer prevention care performance.

## Conclusion

The results of this study reveal that care workers can effectively perform pressure ulcer prevention activities through continuous training and education on pressure ulcer prevention and the provision of an environment that enables extended careers and work experiences. The development of a training program that delivers differentiated and efficient training content that reflects the needs of care workers is necessary rather than a universal and comprehensive education. For effective pressure ulcer prevention in long-term care facilities, appropriate training that can cultivate and expand knowledge related to pressure ulcer prevention of care workers is required as care workers account for the largest number of personnel at such facilities.

Pressure ulcer prevention should be individualized at the care worker level because care workers perform direct patient care for the longest time. Their knowledge significantly affects the performance and patient outcomes of pressure ulcer prevention; therefore, essential knowledge of pressure ulcer prevention should be disseminated officially and informed well for all institutions in charge of elderly care. Official guidelines should elucidate what day-to-day tasks should be performed for care workers and what activities should be performed for the management and supervision of pressure ulcer and its prevention by nurses or nursing assistants.

The following proposals are presented from the results of this study.

First, this study determined the relationship between the prevention work of pressure ulcers and prevention knowledge of care workers at long-term care facilities; thus, the development of a basic training and education program about pressure ulcer prevention for care workers is proposed.

Second, the development of standardized protocols for care workers working in long-term care facilities to prevent pressure ulcers and establishment of a national framework for the management of such protocols to facilitate their application and implementation in the tasks of long-term care facilities are necessary.

Third, a replication study of a sample group that can represent Korea’s long-term care facilities must be carried out for a more intricate determination of the impact of pressure ulcer prevention related knowledge and attitude of care workers at long-term care facilities on care performance.

Finally, a future study must be conducted to verify the effects and whether a pressure ulcer prevention program for care workers actually contributes to reducing incidences of pressure ulcers for the elderly.

## Data Availability

The data are not publicly available due to privacy protections and restrictions from the institutional policy for research ethics and Personal Information Protection Act in the Republic of Korea. However, the data can be requested if someone has a reasonable purpose. Please contact the first author, S.L.
